# Progress in Bacteriology

**Published:** 1902-06-14

**Authors:** 


### Progress in Bacteriology.

**Typhoid Fever. Widal's Reaction.**—The occurrence of a partial Widal's reaction is a constant source of trouble to the bacteriologist and may lead to error in diagnosis. Wilson,[1] treating of this matter, describes the partial reaction as follows:—There may be many clumps, but these, as compared with the complete reaction, are more compact in the centre, with the bacilli at the edge of the clump attached by one end only; the centre of the clump often appears to be a fragment of *débris*, and there are many freely-moving forms; the air at the edge of the drop still attracts the organism, which are consequently found there in considerable numbers. The presence of dirt on the cover-glass or of dead or non-motile organisms may give rise to spurious clumps, but if in any focal plane there can be found a field free from clumps the result must not be returned as positive. Of 1,650 cases examined, 34 only gave a reaction on the second examination, and eight cases which were not enteric fever gave a positive result. Of 1,115 negative results 117 were proved to be typhoid, but none of these were tested more than once. Partial reactions were most frequent in tuberculosis and malaria, and were found in less than 7 per cent. of true typhoid cases. A partial reaction, therefore, is of no value to the clinician beyond pointing to the necessity of sending a second specimen. Robin [2] advises that, in order to avoid spurious clumps, 18-hour agar cultures be used. A small quantity of broth is added to the agar culture and enough of the growth scraped off to produce a uniform cloudiness, and from this the hanging drop is prepared. If the dilution employed be 1 in 20 the reaction should be complete in 15 or 20 minutes. If a considerable number of bacilli be found motile, or, if dead, fail to arrange themselves in clumps, the reaction is negative, even although some clumps may have formed. The general accuracy of the reaction may be judged of by the figures recently given by Tobiesen.[3] Of 350 undoubted clinical cases of enteric fever (tested with dilution of 1 in 50), 329 were positive, 17 gave a reaction only with a dilution of 1 in 10, whilst two reacted only with 1 in 5, and two gave no reaction. Of 151 cases other than typhoid fever four gave positive reactions with dilutions of 1 in 25; of 61 healthy persons one gave a reaction with a dilution of 1 in 25. It is rare for the reaction to occur only during a relapse, and the strength of the reaction bears no relationship to the prognosis. Lacquépée[4] has shown that not only does the blood of a patient suffering from typhoid fever agglutinate cultures of Erberth's bacillus derived from himself, but that it also may agglutinate cultures of B. coli derived from the same patient's intestine. Different varieties of coli derived from the same source behave very differently, some agglutinating readily, others with great difficulty. A serum from one typhoid patient may agglutinate cultures of B. coli derived from another patient. These facts indicate that persons suffering from enteric fever have also a superadded coli intoxication. In differentiating between Erberth's bacillus and the B. coli implicit reliance cannot be placed on the agglutination reaction. From the foregoing facts it is evident that there is great need of greater uniformity of technique in obtaining Widal's reaction. Rosenberger[5] draws attention to this important point, and advocates (1) the use of uniform dilution (1 in 50); (2) a definite time limit; (3) an agreement as to what constitutes a positive reaction; (4) the use of a culture of definite age (an incubated culture 24 hours old); (5) a decision as to whether dried blood, fresh blood or serum is to be used; (6) a stated number of tests to be used in each case. He recommends that results shall always be recorded as positive or negative, and the terms "doubtful" or "pseudo" reaction be dropped. The exact cause of agglutination was thought by Nicolle to be due to the coagulation and coalescence of the external layers of the microbes. This theory has been confirmed by Harrison,[6] who by using pyocyanase succeeded in dissolving off the external coating of the bacteria, then finding that even with the most powerful sera the agglutinating property was lost.

[1] Med. News, N.Y., July 20, 1901. [2] Jour. App. Micr., Aug. 1901. [3] Zeit. f. Klin. Med., Bd. 43, Hf. 1–2. [4] Presse Med., June 8, 1901. [5] Amer. Med. Phil., May 1901. [6] Cent. f. Bakt., July 31, 1901.

